# Cranial Surgery

**Published:** 1894-05-19

**Authors:** 


### Progress in Surgery.

### CRANIAL SURGERY.

*Trephining.*—In opening the cranial cavity some surgeons now reflect in one flap the soft parts with the bone adherent to them, as is done in Hartley's operation for removal of the gasserian ganglion.[1] It is held that larger areas of bone may thus be removed with safety, and that as the bone is not detached from the pericranium its nutrition is better preserved and repair made certain. Dr. Pyle[2] describes the operation in detail, and advocates for division of the bone the use of a graduated set of instruments, which he calls diamond drills, in preference to the hammer and chisel. These drills fit into a ball-shaped handle, which is held in the palm of the hand, and "the bone is divided by repeated strokes in the same manner that a wood engraver cuts a deep furrow in a piece of wood." Victor Horsley[3] does not approve of this osteoplastic method. He considers that the pressure necessary in dividing the bone is apt to produce shock by increasing intracranial pressure. He believes that the pericranium has no osteogenetic power, and therefore it is a matter of no great importance whether it is retained in contact with the bone or not. Macewen supports him in this view, and holds that bone is only formed from preexisting bone cells. Horsley recommends that where bone is to be replaced after trephining large portions of bone should be returned rather than that it should be returned in small pieces. In replacing large areas of bone, in order to avoid necrosis and complications and to provide abundant drainage, Macewen considers it is advantageous to drill a series of holes through the slab of bone. Dr. Weir[4] does not reimplant the bone in operating for tumours, or other conditions likely to recur with increase of intracranial pressure.

Kehr[5] has successfully closed, by transplanting a disc of bone, a large opening in the left parietal bone, resulting from a compound fracture five years previously. Depressed fragments were removed at the time of the accident, and paralysis of the right arm and frequent epileptiform seizures had been present afterwards. He raised by chisel a disk composed of the outer table of the bone close to the opening. He retained its attachment to the pericranium and skin by a broad bridge, and moved it along till it closed the defect, and fixed it in position by sutures. Good recovery followed, and two months after the operation there had been no convulsions, and power was return- to the arm.

*Epilepsy.*—In a paper on "Brain Localization in Epilepsy," Dr. Hadra[6] advises the use "of the induced current not solely to find the physiological focus belonging to the muscles giving the initial signal, but to find the spot from where an epileptic attack of the same nature the patient is accustomed to suffer from can be started independently of the fact whether those two points coincide or not." He contends there is no proof that the focus of the muscles giving the initial signal is really the diseased portion of the cortex. After the brain surface has been exposed, if no definite lesion is visible, electrodes, as devised by Keen, consisting of two wire points, a quarter to half-an-inch apart, should be applied over the whole area. When the place from which the epileptic convulsions can be elicited is found, the experiment should be repeated so as to leave no doubt about it. If excision is performed the current then has to be applied to the borders of the brain wound again and again till epileptic symptoms can no longer be produced, to ensure that the diseased tissue has been thoroughly removed. In support of his views he gives details of three operations. Dr. McCosh[7] thinks that, considering the great advances in the localization of cerebral functions, the results from operation for the cure of true idiopathic epilepsy are rather disappointing. They are much more satisfactory in connection with Jacksonian epilepsy. He gives details of three operations followed by excellent results. The fits were due to pressure from extradural hæmorrhage in two cases and from an organized clot in the third. Dr. Carline[8] records a case of successful trephining for traumatic epilepsy.

*Cerebral Tumour.*—According to Mr. Horsley, in the treatment of cerebral tumours the appeal to surgical aid is, as a rule, made too late, when, in fact, the case is beyond cure by operation and often beyond palliation. Medical treatment should not be continued too long; Starr says not longer than three months, Horsley not longer than six weeks, if there is no marked improvement. It must be remembered that moderate improvement under large doses of iodide of potassium is no proof that the tumour is gummatous, for in most tumours of the brain moderate relief may result for a time. Horsley and Macewen[9] strongly advocate palliative operations in connection with cerebral tumours, holding that even although a cure cannot be looked for, the relief of the headache, optic neuritis, and vomiting, which usually follows the lessening of intracranial pressure, more than justifies the operation. They recommend that the operation for the removal of tumour be performed in two stages, *i.e.*, removing of bone on one day, and then a few days later opening dura mater and removing tumour. This lessens the risk of shock, and the soldering of the membranes which is allowed to take place shuts off the subdural space and prevents blood, &c., escaping into it. The relief which may follow the removal of an area of bone was well shown in a case of acromegaly in which Dr. Caton[10] diagnosed enlargement of the pituitary body. There was no hope of removing the tumour, but with a view to relieving the vomiting, intense pain, and advancing blindness, Mr. Paul operated. Three square inches of bone were removed from the temporal fossa and the dura mater was allowed to bulge out freely. Nothing further was done, and the soft parts were closed over the opening. The patient lived for three months and was entirely free from vomiting and pain. The blindness was not checked. After death a large sarcoma of the pituitary body was found. Operations for the removal of cerebral tumour have recently been recorded by Keen,[11] Pel,[12] Richardson and Walton.[13] Clarke[14] gives an interesting report of a case of intracranial hydatids.

*Encephalocele.*—Mayo[15] has excised with recovery a congenital, pulsating encephalocele, which protruded from the occiput of a child eighteen months old. It communicated with the right lateral ventricle, and was as large as an orange. A fatal case is reported by

Dr. Mackie,[16] of Elgin. Three days after the birth of the child he removed an encephalocele from the occipital region, as there was ulceration of its covering. Thirty-seven days later hydrocephalus appeared and advanced rapidly, the child dying fifty-seven days after operation.

*Cranial Drainage in Tubercular Meningitis, &c.*—Dr. Ord and Mr. Waterhouse[17] record a case diagnosed as tubercular meningitis, which recovered after drainage of the subarachnoid space. The child of five years old had all the symptoms and history of tubercular meningitis. As extreme pain and marked optic neuritis pointed to intracranial pressure, and as there were signs of approaching coma, it was decided to operate with a view to relieving pressure. A disc of bone was removed midway between the external occipital crest and the mastoid process. The dura mater bulged out. It was incised, and also the arachnoid, a few drops of greenish serum escaping. The cerebellum bulged into the opening. A probe bent at right angles near the point was passed between the cerebellum and arachnoid to the falx cerebelli, and was then rotated so that the point passed into the subarachnoid space between the medulla and cerebellum. A drainage tube was inserted and left in. The bone was packed in small fragments round the tube, which was brought out through an opening in the skin flap. Serous fluid continued to drain away till the tube was removed twenty days after operation. The child made an excellent recovery, and in a month seemed quite well.

While making an exploratory trephining in a case with well marked pressure symptoms, McCosh[18] was led from the absence of tumour or abscess to suspect that the extreme pressure was due to fluid in the ventricle. He therefore tapped the right ventricle, and withdrew an ounce of clear fluid. Drainage was kept up for forty-eight hours. Recovery was perfect. No definite cause was discovered.

Professor Wyss[19] recommends tapping of the skull, or else of the sub-dural space in the lumbar region, in the following circumstances—(1) Where there are symptoms of great pressure in fresh cases of meningitis; (2) in the later stages of new tubercular meningitis, and in congenital syphilitic hydrocephalus; (3) in swelling of the head after recovery from meningitis; (4) in loss of sight from hydrocephalus when there is no neuritis.

*Craniectomy in Idiocy.*—Most surgeons believe that only a small number of the conditions causing idiocy in children are amenable to operative interference, and all admit the difficulty of diagnosis.

Bourneville,[20] after studying all the recorded cases of craniectomy, holds that the results obtained by the majority of surgeons are slight, doubtful, or negative, and that, further, there is the risk of paralysis, convulsions, and death following the operation. As a rule, no premature ossification of the sutures exist, and by present methods of investigation the condition cannot be diagnosed. He considers that better results are to be obtained by attention to the general health and a lengthened course of training on the lines laid down by the elder Seguin.

*Removal of Gasserian Ganglion.*—In operations for intractable neuralgia of the fifth nerve performed lately the method followed most frequently has been that of Dr. Hartley. This consists in refecting a flap containing skin, muscle, pericranium, and bone, and exposing a circular area of dura mater about three inches in diameter. The base of the flap is at the zygoma, and its apex at the supratemporal region. The dura mater and the brain are raised by broad retractors, and the foramen rotundum and foramen ovale exposed to view, with the second and third divisions of the fifth nerve. These branches are divided at the foramina, and the part between these and a point beyond the Gasserian ganglion is excised. To prevent, if possible, any reunion the ends of the nerves are pushed through the foramina.

Dr. Tiffany[21] has recently operated by this method on four cases, and in three of them seen since operation there has been no return of the pain. Dr. Finney[22] records two successful cases, and one which ended fatally from heart failure and shock. Dr. O'Hara has had a successful case. Dr. Baker[23] performed Rose's operation on a man fifty-two years of age. The patient died of shock.

*Skin Grafting on Denuded Cranium.*—When extensive surfaces of the cranium have been denuded, Thiersch's method of applying large grafts, after healthy aseptic granulations have been obtained, proves very successful.

Riegner[24] has recently successfully treated a case of complete denudation of the cranium by this method. Dr. Pond[25] also had a case which did well till the patient died of diphtheria. Pond saw the case twelve days after injury; the whole cranium was almost stripped of pericranium, and the wound was septic and without granulations. To procure granulations he rendered the parts aseptic and then made seventy-five perforations through the outer table of the skull into the diploë. In eleven days granulations appeared through these openings and spread over the bones. On the granulating surface thus obtained grafts were placed and did well.

[1] Annals of Surgery, Jan., 1894. [2] Medical Record, Feb. 10, 1894. [3] Brit. Med. Journ., Dec. 23, 1893, p. 1367. [4] Inter. Clinics, vol. iii., 1893. [5] Brit. Med. Journ., Dec. 30, 1893. [6] Annals of Surgery, March, 1894. [7] Amer. Journ. Med. Sci., March, 1894. [8] Brit. Med. Journ., Feb. 24, 1894, p. 408; [9] Brit. Med. Journ., Dec. 23, 1893, p. 1367; [10] Brit. Med. Journ., Dec. 30, 1893, p. 1421. [11] Amer. Journ. Med. Sci., Feb., 1894. [12] Centralblatt für Chir., No. 9, 1894. [13] Lancet, Feb. 24, 1894, p. 494. [14] Amer. Journ. Med. Sci., March, 1894, p. 333. [15] Centralblatt für Chir., No. 9, 1894. [16] Brit. Med. Journ., March 10, 1894, p. 510. [17] Lancet, March 10, 1894, p. 598. [18] Amer Journ. Med. Sci., March, 1894. p. 239. [19] Provinc. Med. Journ., Feb. 1, 1894. [20] Amer. Journ. Med. Sci., Feb., 1894, p. 232. [21] Annals of Surgery, Jan., 1894. [22] Brit. Med. Journ., Dec. 23, 1894, Ep. p. 101. [23] Amer. Journ. Med. Sci., March, 1894. [24] Provinc. Med. Journ., Feb., 1894. [25] Med. Record. Dec. 16, 18[illegible]3.

